# Experimental investigation on rockburst behavior of the rock-coal-bolt specimen under different stress conditions

**DOI:** 10.1038/s41598-020-64513-3

**Published:** 2020-05-05

**Authors:** Gen-shui Wu, Wei-jian Yu, Jian-ping Zuo, Chun-yuan Li, Jie-hua Li, Shao-hua Du

**Affiliations:** 1 School of Mechanics and Civil Engineering, China University of Mining and Technology, Beijing, 100083 China; 2 State Key Laboratory of Coal Resources and Safe Mining, China University of Mining and Technology, Beijing, 100083 China; 3 School of Resource & Environment and Safety Engineering, Hunan University of Science and Technology, Xiangtan, Hunan 411201 China; 4 Department of Civil Engineering, Monash University, Clayton, Victoria 3800 Australia

**Keywords:** Civil engineering, Natural hazards, Coal, Petrology

## Abstract

Coal and rock burst are one of the main dynamic disasters that affect coal mine production. In this paper, the burst structural model of the rock-coal-bolt (RCB) system and the burst tendency criterion are established on the background of deep thin coal seam mining. Uniaxial and triaxial mechanical tests under different stress states are carried out on RCB specimens with different angles. Combined with thermal imaging, the mechanical behavior of the inclined RCB specimen under uniaxial loading is discussed. The results show that the burst tendency of the RCB specimen increases with the angle. The stress-strain curves of some uniaxial and triaxial test specimens show two or more peaks, and the thermal imaging evolutionary process shows that the cracks of the coal and rock develop from shear to tension shear cracks. There is a further development of fracture and energy accumulation between the first and second peaks in the stress-strain curve of the specimen. Therefore, the failure degree of the second peak of the specimen may be stronger than that of the first peak. Additionally, the established stiffness coefficient and burst energy index can better describe the burst tendency of the RCB specimen under different stress states. The results show that the burst tendency of the RCB specimen under the triaxial test is much higher than that of the uniaxial test. In other words, it also explains that the essence of the burst failure of the surrounding rock in the roadway is the initial instability induced by the inside surrounding rock in the roadway. Moreover, the burst tendency is the largest when the rock and coal combination angle is 15°, and the burst damage range may also be increased by the failure of internal coal and rock mass.

## Introduction

**Figure 1:**
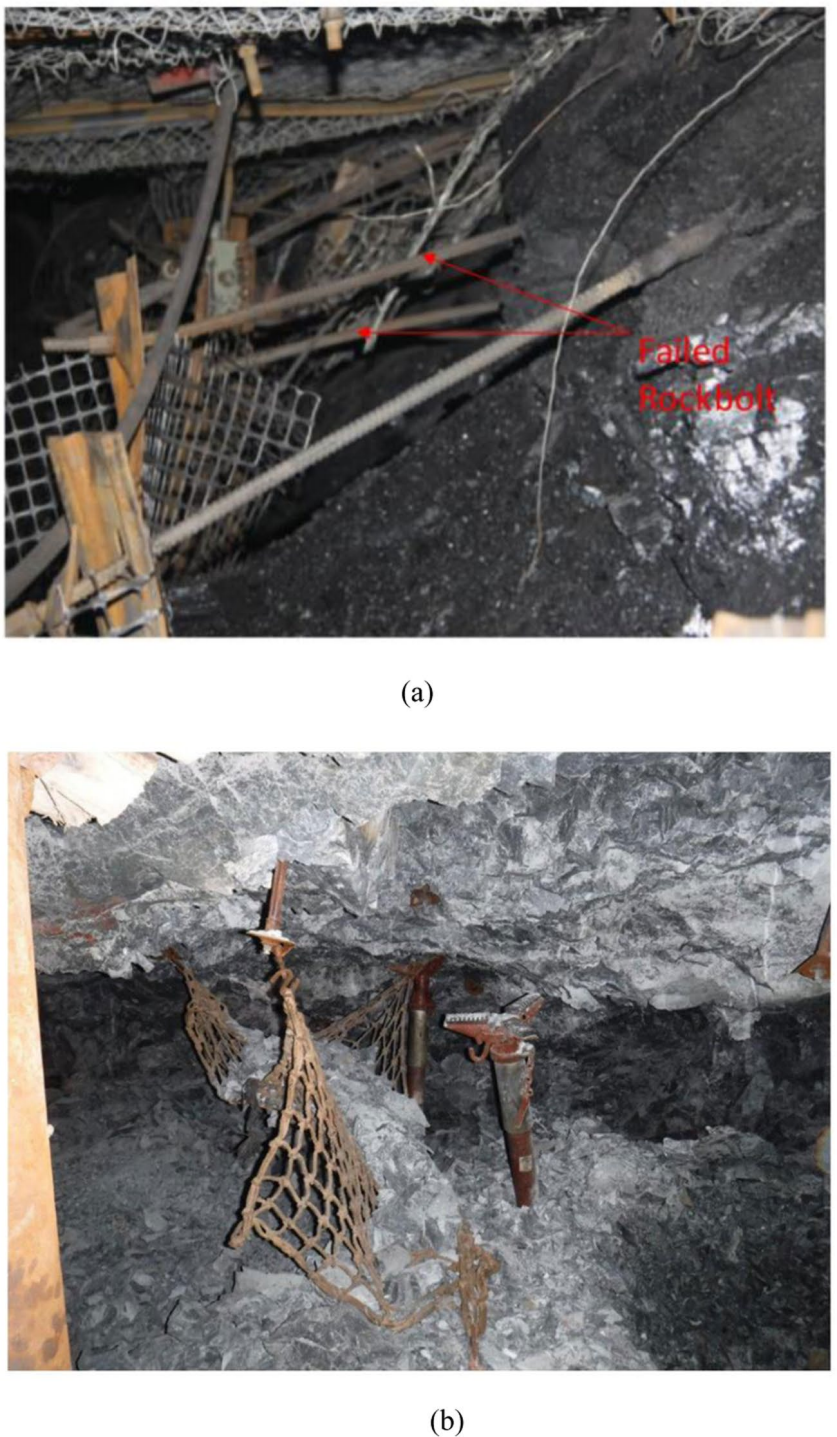
Typical failure mode of rock burst (**a**) Failure of coal rock mass and support^12^; (**b**) Failure of retaining elements during rockburst (South African metal mine^15^).

Rock burst is one of the main coal and rock dynamic disasters that affect coal mine production. This kind of burst disaster refers to the sudden, sharp and violent release of elastic energy in the coal rock mass when the coal and rock combine system reaches the ultimate strength. Generally, when the rockburst occurs, the coal and rock mass and support structure are suddenly destabilized and damaged, causing casualties, roadway and equipment damage. Rockburst behavior is mainly related to high ground stress, far-field mining stress disturbance, faults and “coal seam-roof and floor-support structure” structure, and the mechanism of strata movement is complex^1–6^. Different from the conventional mine pressure behavior, rock burst damage is induced by the free space from the internal coal and rock to the roadway. And the fracture development of the internal coal and rock mass is earlier and faster than that of the external surrounding rock. Due to the unpredictable nature of burst damage, it is very difficult for producers to master and predict the law of occurrence of coal burst. Strong rock burst in coal mines will lead to severe roadway closure and equipment damage, causing personal injury and significant property damage^7–11^. As shown in Fig. 1(a), rock burst failure in Yima coalfield (a Chinese coal mine) has resulted in serious deformation and displacement of the coal wall in the roadway, the bolt was pulled out and the coal body collapsed^12^. This kind of strong rock burst is also common in many deep mines in South Africa^13,14^. Figure 1(b) is shown an example of a weak retaining element (movable net) failure during a rock burst. The supporting structure cannot prevent the occurrence of an instant rock burst^15^. With the continuous increase of mining depth, more and more underground coal mine roadway projects are threatened by a rock burst. Therefore, in the past 40 years, many scholars have begun to pay attention to the occurrence mechanism of rock burst, classification of burst indicators, monitoring and early warning technology of rock burst, anti-impact technology and other aspects, and have achieved many remarkable results^16–22^. Many researchers regard the “roof-coal-floor” of the deep coal mine roadway as an integrated system, simplifying the structure of coal seam and surrounding rock into a sample of rock and coal with bonded or frictional interface. They mainly focus on this system used laboratory test or mechanical properties in numerical simulation methods^23–34^. For instance, Zuo *et al*.^28,29^ and Chen *et al*.^30^ conducted a large number of experimental studies on the failure mechanism and mechanical properties of rock-coal (RC) specimen, and explained the burst tendency of RC system due to mechanical differences and their non-compliance linear failure characteristics. Zhao *et al*.^31^ built an equivalent homogeneous model of the RC specimen. They assessed the effects of interface cohesive strength, rock thickness, and stress level on the failure behavior of the combination model. Results demonstrate that the proposed model reflects the strength behavior of a more complex model composed of different rock mediums and structural plane. Chen *et al*.^27^ performed uniaxial compression tests (UCT) on samples of coal-oil shale and analyzed the strength, macroscopic failure initiation (MFI), and failure characteristics of the specimens. Huang and Liu^32^ conducted UCT on RC specimens at different loading rates to accurately evaluate the danger of rock burst during coal mining. To study the occurrence process and mechanism of strained rock burst in deep circular caverns under high-stress, Gong *et al*.^33,34^ conducted simulation experiments under four different three-dimensional stress states on the cubic granite samples with preformed circular holes using true triaxial electro-hydraulic servo mutagenic test system. Moreover, many researchers have also concentrated on a series of analyses of the response of impact stress on the reinforcement and instability of surrounding rock support structures^35–40^. The process of RC body failure can be seen as a result of the accumulation and release of energy inside the coal-rock, and this process is completed in a very short time. Energy release patterns of coal rocks include the sound of rock fractures and sudden temperature changes at the time of failure. Rock energy dissipation is irreversible and non-linear, which is the root cause of irreversible non-linear damage and fracture of coal and rock mass^41–43^. Particularly, the brittle coal body has a higher burst tendency after high-stress loading.

From the above research content, most researchers pay more attention to the horizontal RC system and less consider the burst tendency behavior of inclined RC system. As we know, reserves of inclined thin coal seams in China are very large, and the inclined coal seams larger than 35° account for more than 20% of the total reserves of coal resources in China. Furthermore, with the continuous increase of coal mining depth, thin coal seam resources widely distributed in South China are facing the problems of complex geological structure and large change of coal seam angle^44–48^. As a result, mining thin coal seam in complex conditions, the accident rate of rock burst disaster is increasing. Additionally, from the perspective of the site (Fig. 1), burst failure is not simply a mechanical behavior of coal and rock system, but also has some connection with the supporting reinforcement structure. Therefore, in this paper, a structural model of the RCB burst system and burst stiffness index are constructed, and a series of uniaxial and triaxial compression tests (UCT and TCT) are performed on the rock-coal-bolt (RCB) specimen with different angles. The relationship between macro fracture evolution and the stress process in UCT is compared. Finally, according to the proposed burst stiffness coefficient and burst energy index, the burst tendency of RCB specimens under different stress states is analyzed.

## Simplified description of rock-supporting structure and laboratory test method

### Burst induction mechanism of rock-coal-bolt (RCB) system

**Figure 2:**
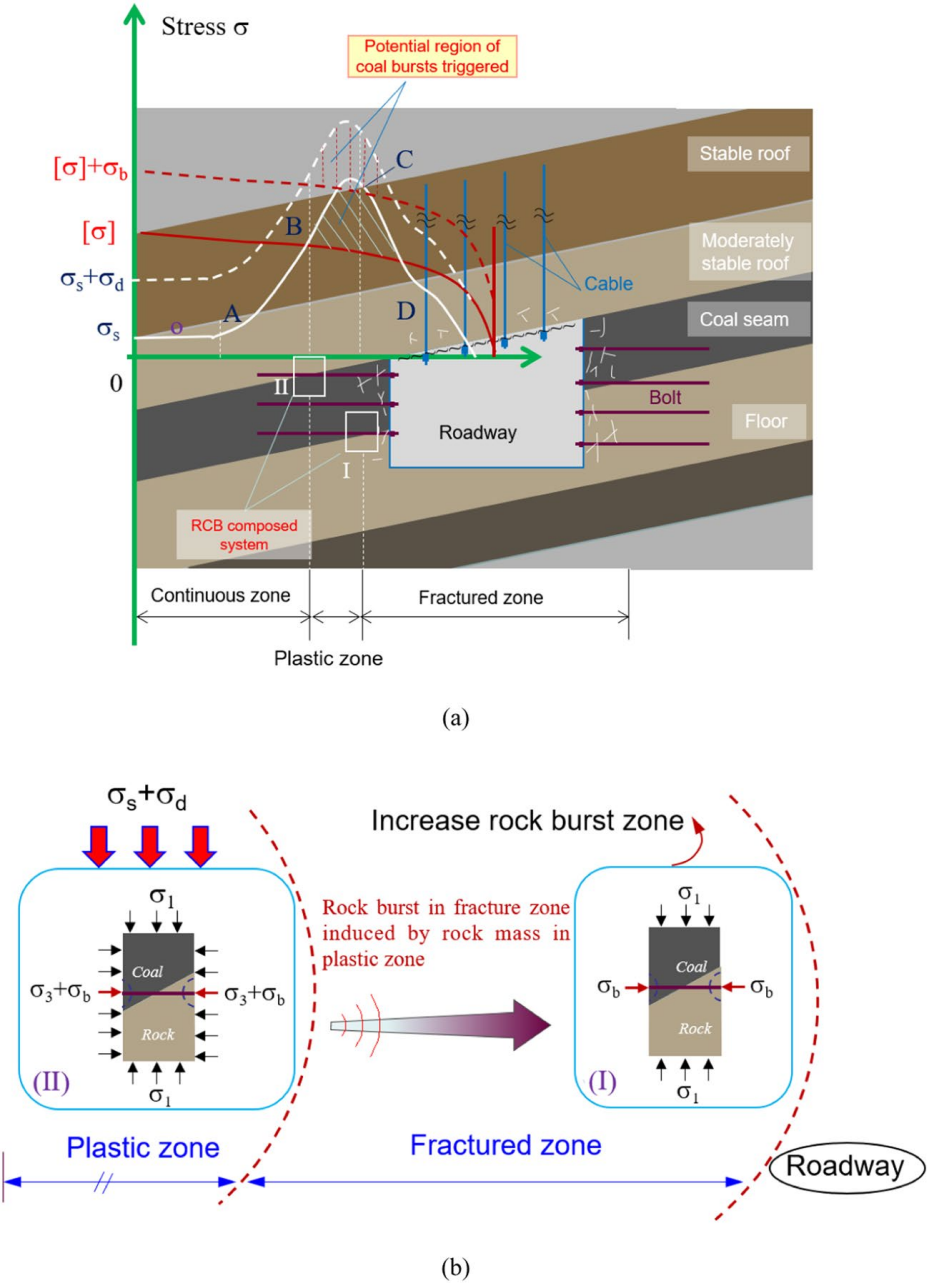
Mechanism of surrounding rock-support burst induction and simplification of surrounding rock stress (**a**) Division of surrounding rock and stress distribution gradient of roadway; (**b**) Stress state simplification of inclined RCB system.

The stress of the roadway surrounding rock is redistributed with thin coal seam mining, forming a stress-enhancing zone from inside to outside. Figure 2(a) indicates the surrounding rock partition structure and stress distribution gradient of the roadway. According to the stress distribution characteristics, the surrounding rock of the roadway can be divided into residual stress zone, plastic zone and elastic continuous zone^49^. Among them, the surrounding rock in the residual stress zone is within the post-peak residual stress (CD) of the rock. The boundary between the residual stress zone and the plastic zone is the peak stress point (point D) of the rock, and the BC section corresponds to the plastic yield zone of the surrounding rock. Before the point B of the rock damage stress is the continuous elastic zone. The solid red line in Fig. 2(a) is the strength [σ] of rock and coal without support. If the strength [σ] of rock and coal intersects with the σ_s_ curve of surrounding rock under static load, the intersecting area will cause the coal and rock mass to be destroyed and release elastic strain energy, which may induce rockburst (i.e., σ_s_ > [σ]).The red dotted line in Fig. 2(a) is the strength of rock and coal after supporting ([σ]+ σ_b_). The support reinforcement increases the strength of the rock and coal, reduces the intersection area with the strength σ_s_ of the surrounding rock, and further reduces the possibility of damage of the rock and coal with the support. However, the redistribution of regional stress caused by coal mining may lead to far-field fault slip or roof rupture and related microseismic (MS) events, resulting in additional dynamic stress σ_d_. At this time, due to the increase of dynamic stress, the stress peak value (σ_s_) will increase, causing the peak point to transfer to the interior of the surrounding rock. Then, the range of stress increasing area and burst zone will expand, forming the strength (σ_s_ + σ_d_) as shown by the white dashed line in Fig. 2(a). Under such a stress environment, rockburst is easy to occur as long as the superposition of static stress and dynamic stress (total static stress σ_s_ + σ_d_) exceeds the strength of supporting coal and rock mass (i.e., σ_s_ + σ_d_ > [σ]+ σ_b_).

Figure 2(b) shows the simplified stress characteristics of rock and coal units in zones I and II. These two states are located between the plastic zone and the residual stress zone. The main difference is that the RCB system unit in area I is approximately uniaxial without lateral pressure. In contrast, the RCB system unit in area II is under three-dimensional stress and has confining pressure.

### Criterion of rock burst tendency of RCB specimen

**Figure 3:**
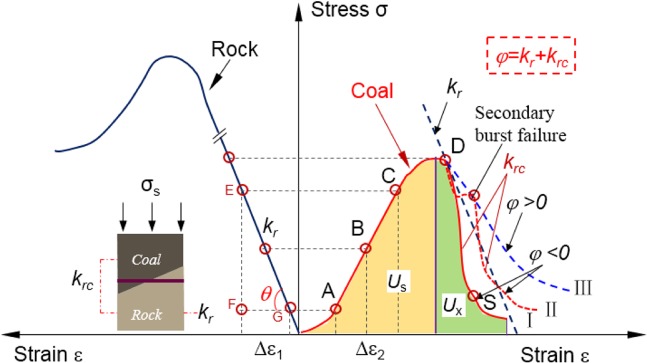
Burst failure model and burst stiffness coefficient of coal body in the RCB system (revised from^44^).

Figure 3 shows a model of the coal burst failure stiffness of the RCB system under static load^49^. In the roof-coal-floor system, coal is assumed to be a fractured or softened material with non-linear behavior. The left side of Fig. 3 describes the stress behavior of the surrounding rock under loading, and the stress behavior of the RCB system is on the right. The roof, coal, and floor are considered as a unified surrounding rock system. If the rock has much greater stiffness and strength than coal, the coal’s stress behavior can be used instead of the RCB system. As shown in Fig. 3, the stiffness of the rock is the slope before the peak of the rock (*K*_r_), and the stiffness of the coal and rock has two stages, namely the stiffness before the peak and drop stiffness after the peak (*K*_rc_). The equation of rock mass stiffness coefficient *K*_r_ is as follows:

1 $${K}_{r}=\,\tan \,\theta =\frac{EF}{FG}$$

Assuming the coal is tightly bound to the surrounding rock, the strain changes (Δε_rc_) in the coal will simultaneously cause deformation of the surrounding rock (roof and floor). The surrounding rock strain (Δε_r_) can be expressed as:

2 $${\Delta {\rm{\varepsilon }}}_{{\rm{r}}}=\frac{{K}_{{\rm{rc}}}}{{K}_{{\rm{r}}}}{\Delta {\rm{\varepsilon }}}_{2}$$

where *K*_r_ is the stiffness of the surrounding rock; *K*_rc_ is the stiffness of the coal. The strain of the entire coal rock system is:

3 $${\Delta {\rm{\varepsilon }}=\Delta {\rm{\varepsilon }}}_{{\rm{r}}}+{\Delta {\rm{\varepsilon }}}_{{\rm{rc}}}=\frac{{K}_{{\rm{r}}}+{K}_{{\rm{rc}}}}{{K}_{{\rm{r}}}}{\Delta {\rm{\varepsilon }}}_{{\rm{rc}}}$$

The ratio of coal strain to total strain can be written as:

3 $$\frac{{\Delta {\rm{\varepsilon }}}_{{\rm{rc}}}}{\Delta {\rm{\varepsilon }}}=\frac{1}{1+\frac{{K}_{{\rm{rc}}}}{{K}_{{\rm{r}}}}}$$

Under the static load, the total stiffness coefficient of the RC specimen and rock can be used to quantitatively describe the burst behavior of the RCB system to reflect the process of coal and rock from stability to instability (see Fig. 3), as shown in Eq. (4):

4 $$\varphi ={K}_{r}+{K}_{rc}$$

The total stiffness coefficient of the RCB system contains the following process:

1. From the elastic stage to the pre-peak failure stage (AC), *φ* > 0, where *K*_rc_ and *K*_r_ > 0; At this stage, the surrounding rock and coal are both in the elastic energy storage state, which is the calm period before the failure of coal. In other words, when the coal starts to convert elastic energy into plastic deformation, the surrounding rock is still accumulating elastic strain energy. It can be observed Eq. (3) that the ratio of coal strain to total strain (Δε_rc_ /Δε) increases with the decrease of *K*_rc_*/K*_r_, and the essence of this change is that microcracks in coal begin to develop and expand.
2. The RCB system gradually reaches the residual strength stage, i.e., *φ* > 0; where *K*_r_ > 0, ${K}_{rc}\le 0$. At this time, the failure process of the RCB system is static or metastable.
3. In the residual stage of brittle failure, the strength of the RCB system suddenly changes, i.e., *φ* < 0; where $|{K}_{rc}| > |{K}_{r}|$ and *K*_r_ < 0. At this stage, the stress of the RCB system declines with the gradual loss of its bearing capacity, and *K*_rc_ becomes negative. At this stage, *φ* = 0 is the critical value of the rock burst, the roof-coal-floor system can reach an extremely unstable state, i.e., Δε_rc_/Δε→∞. At this time, a dynamic failure event will be triggered, corresponding to the occurrence of the rock burst.

The international main indexes for evaluating the risk of rock burst include burst energy index, elastic energy index and dynamic failure time^32,50,51^. The burst energy index classification method of the coal is also stipulated in the national standard of the people’s Republic of China GB/T 25217.2-2010. The burst tendency of RCB system can be evaluated by determining the burst energy index *B*_E_ and the uniaxial compressive strength (UCS) of coal according to the stress-strain curve (Fig. 3).

5 $${B}_{{\rm{E}}}=\frac{{U}_{{\rm{s}}}}{{U}_{{\rm{x}}}}$$

where, *U*_s_ is the deformation energy accumulated in the curve before the peak; *U*_x_ is the deformation energy lost in the curve after the peak. Among them, *B*_E_ < 1.5 has no burst, 1.5 < *B*_E_ < 5 is a weak burst, and *B*_E_ > 5 is a strong burst.

The UCS burst index of standard coal is: UCS < 7 no burst; 7 <UCS < 14 weak burst; UCS > 14 strong burst (unit MPa).

### Preparation and laboratory test method of RCB specimen

**Figure 4:**
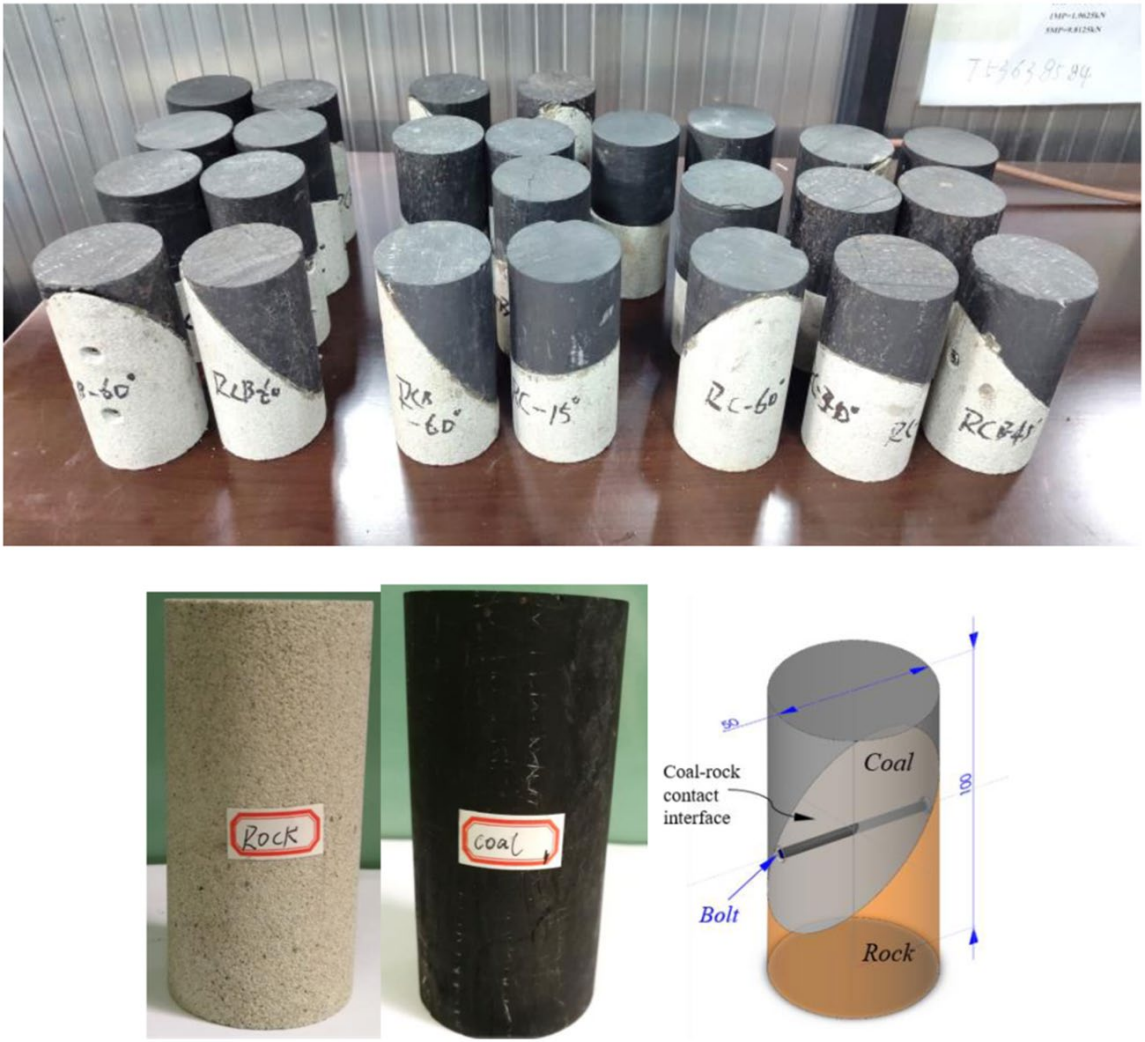
Specimen of sandstone, coal and RCB specimen (partial).

Figure 4 shows the coal, sandstone and RCB specimen of the test. The specimens were taken from the inclined thin coal seam coal mine with a depth of 600 m in Hunan Province, China, in which the rock was sandstone without obvious bedding. Based on the previous experimental research foundation^21,32,52,53^, the coal and sandstone are combined and cemented for a second time according to the volume ratio of 1:1. To better conform to the actual engineering background, there should be a certain strength of the cohesive force between the coal and rock contact surfaces. Therefore, marble glue with good bonding performance and widely used in the geotechnical engineering field is selected to uniformly bond the coal-rock interface.

Four kinds of anchoring specimens with different combination angles are set up. The angles are 15°, 30°, 45°, and 60° respectively (i.e., RCB-15, RCB-30, RCB-45, RCB-60, respectively). The no anchor horizontal RC specimen (RC-0), sandstone and coal are considered as control groups. A horizontal bolt is set for all specimens with combined angles of 15°, 30°, and 45°. To ensure the bolt anchoring effect of the large contact area of the RCB-60 specimen and the reliability of the test, we have set up an additional horizontal bolt to anchor the RCB-60 specimen simultaneously (i.e. the RCB-60 specimen is the anchorage of two horizontal bolts). The bolt is a full-length anchor threaded steel bolt with a diameter of 3 mm and a length of 50 mm, and the tensile strength of the bolt is more than 520 MPa. The anchoring process of the inclined rock-coal-bolt specimen was firstly drilled a horizontally anchor hole with a 3 mm diameter drill. Then, the threaded steel bolt is penetrated through the inclined plane of coal and rock and anchored in full length.

According to the test method recommended by the International Society for Rock Mechanics (ISRM)^54^, the top and bottom ends of the specimens are polished before the test to ensure that the two surfaces are smooth and parallel <0.02 mm. As shown in Table 1, the diameter error of all test combination specimens is 50 ± 1 mm and the height error is 100 ± 2 mm. Additionally, to improve the accuracy of the test, each group of tests was repeated 3 times.

**Table 1:** Specimen Physical parameters (average value).

Specimen	Dimensions(mm)		P-wave velocity (V_p_)/m·s^−1^	Density(kg/m^3^)
	Length	Diameter		
Coal	101.5	49.7	1062.72	1229
Rock	99.4	49.4	1950.98	2271
RC-0	99.5	49.3	1941.93	1750
RCB-15	101.6	49.1	1851.85	1722
RCB-30	101.1	49.6	1539.92	1733
RCB-45	99.5	49.3	1515.15	1701
RCB-60	101.9	49.1	1904.94	1804

**Figure 5:**
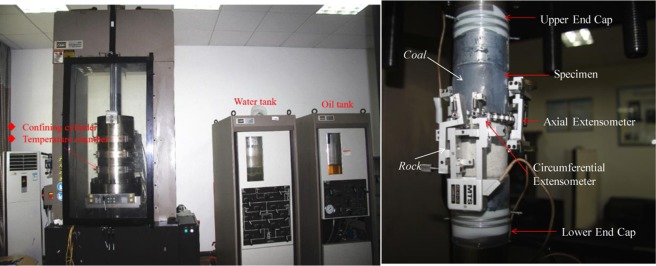
MTS-815 rock mechanics testing system and extensometer configurations.

The tests include UCT and TCT (two different stress state tests), the specific test methods are:

**1. TCT**: Fig. 5 shows the MTS-815 servo-controlled rock mechanics test system manufactured by the MTS company of the United States. The system mainly consists of a loading system, a controller, a measuring system and other parts, and has four independent closed-loop servo control functions of axial pressure, confining pressure, pore water pressure and temperature. The TCT loading scheme is as follows: gradually increase 5 MPa/min to the target confining pressure of 10 MPa, and then keep the confining pressure constant. After the confining pressure is constant for 2 min, control the loading with displacement (0.15 mm/min) until the specimen is damaged.

**Figure 6:**
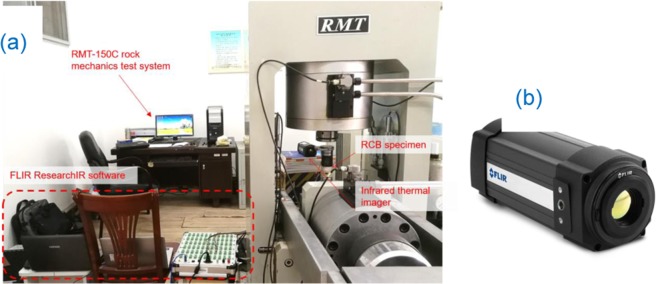
UCT equipment (**a**) RMT-150C electro-hydraulic servo rock mechanics test system (**b**) FLIR infrared thermal image monitoring system.

**2. UCT**: Fig. 6(a) shows the RMT-150C electro-hydraulic servo rock mechanics test system developed by the Institute of Rock and Soil Mechanics, Chinese Academy of sciences. To meet the monitoring conditions of thermal imaging, the UCT scheme is to adopt displacement loading control, and the loading rate is constant at 0.005 mm/s until the specimen is failure.

**3. Infrared thermography monitoring**: The infrared thermography monitoring system is used for synchronous monitoring during the UCT. Figure 6(b) shows a SC-325 infrared radiation remote sensing device manufactured by the FLIR Company of the United States. The specific parameters of the instrument are the noise equivalent temperature difference (NETD) <0.05 °C, image frame frequency 60 Hz, and infrared image resolution are 320 × 240 pixels. To reduce the interference of environmental factors on thermal imaging, the ambient temperature of the atmosphere is recorded before each test. During the test, the curtain of the laboratory shall be pulled up, all the test personnel shall be forbidden to move about. Besides, the testing machine and infrared radiation remote sensing device shall be covered with radiation protection cover until the test is finished.

## Test results of the RCB specimen

### Stress-strain curve

**Figure 7:**
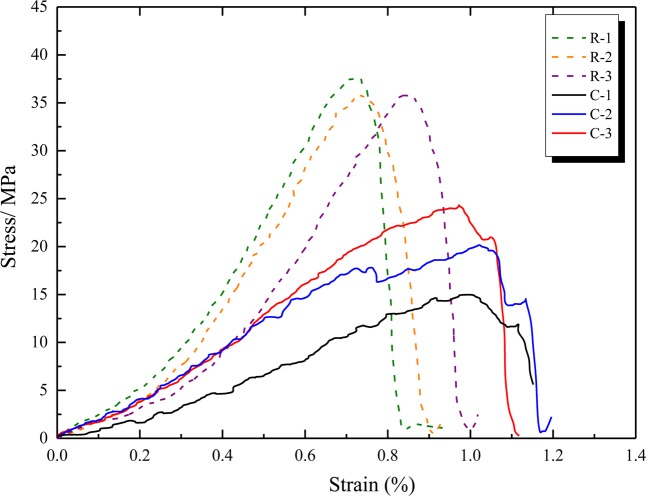
Stress strain curve of coal and sandstone (UCT).

Figure 7 illustrates a stress-strain curve of coal and sandstone (UCT). The average UCS and elastic modulus of sandstone are 37.06 MPa and 11.6 GPa respectively. The average UCS of coal is 20.04 MPa and the elastic modulus is 3.9 GPa. According to the classification of the UCT coal burst index in section 2.2, the coal is determined as a strong burst tendency (UCS > 14 MPa). Repeated tests on the specimens show that the discreteness of the samples has little influence on rock deformation parameters (elastic modulus) (Fig. 7). Because there are some primary fractures in the coal, except for the difference of UCS, the elastic modulus is consistent. Therefore, the typical test results can represent the rock mechanical characteristics of the RCB specimen.

**Figure 8:**
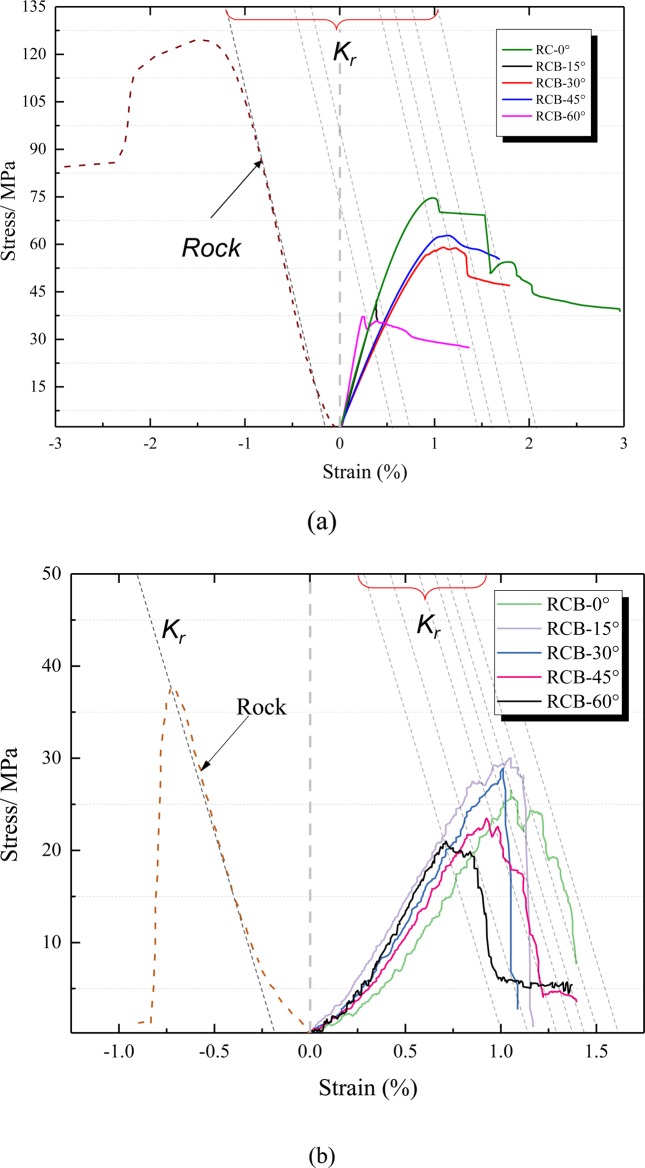
Stress-strain curve of RCB specimen. (**a**) sandstone and RCB specimen (TCT); (**b**) sandstone and RCB specimen (UCT).

Figure 8 illustrates a stress-strain curve of inclined RCB specimen (under TCT and UCT conditions). Under the triaxial stress state (Fig. 8a), the peak axial strain of the RCB-60 specimen is the smallest (0.26%). The smaller the angle is, the larger the corresponding peak axial strain is, and the RCB-30 specimen is the maximum peak axial strain (1.21%) (Fig. 8b). The UCT stress-strain curves of RCB specimen have undergone compaction, linear elasticity and post-peak stages, which are similar to the UCT curves of the sandstone and coal (Fig. 7). The specimen have a large stress drop after the peak of the stress-strain curve and loses a large residual strength. Similarly, the minimum peak axial strain is 0.69% of the RCB-60 specimen under TCT conditions, and the smaller the angle is, the larger the peak axial strain is. The maximum peak axial strain is 1.70% of the RC-0 specimen. The characteristics of the peak axial strain and angle for TCT and UCT show that under the same conditions, the larger the angle is, the smaller the peak axial strain is. Therefore, under the same stress loading condition, the large-angle specimen will fail faster than the small-angle specimen.

### Deterioration of mechanical parameters

**Figure 9:**
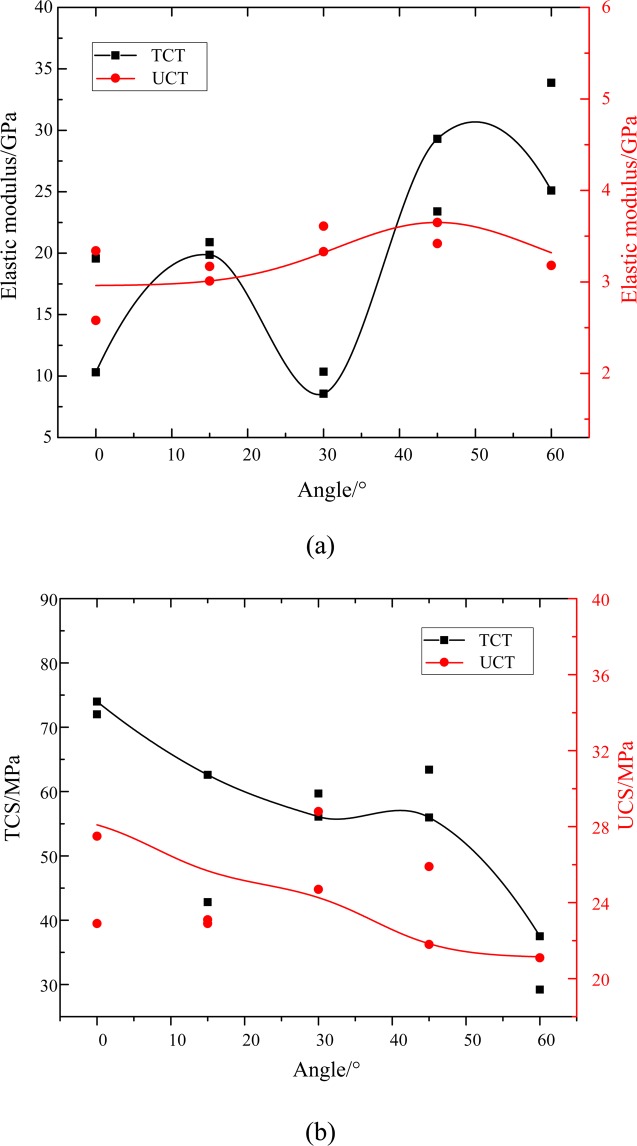
Deformation parameters and mechanical parameters of RCB specimen (**a**) elastic modulus (**b**) peak strength.

Figure 9 shows the relationship between the deformation parameters, mechanical parameters and the angle of the RCB specimen (under UCT and TCT conditions). In the UCT, the elastic modulus of the RCB specimen increases first and then declines with the increase of angle, and the change range remains relatively stable, within 2.5~4.0 GPa. In the TCT, the elastic modulus of the specimen reflects the amplitude change of the wave, and the whole cycle is increasing first and then decreasing. The elastic modulus varies widely with a maximum value of 33.6 GPa and a minimum value of 8.1 GPa (Fig. 9a). The RC-0 specimen has the highest peak strength (74.8 MPa for TCS; 54.1 MPa for UCS). With the increase of the angle, the UCS and TCS decreased to some extent, showing a linear relationship (Fig. 9b). On the whole, the RCB specimen deformation parameters and mechanical parameters of TCT are larger than those of UCT.

### Thermal image evolution (UCT)

**Figure 10:**
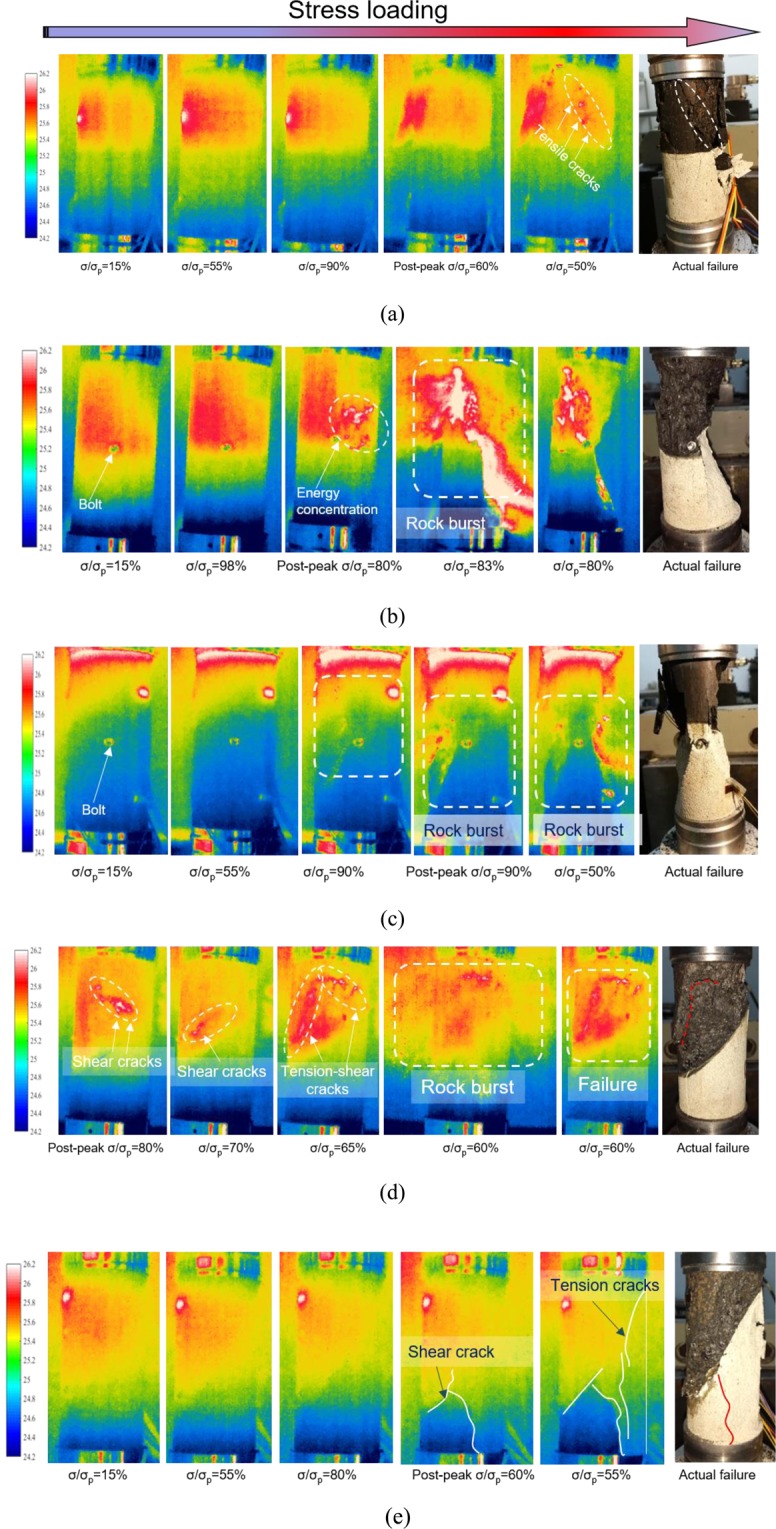
Thermal image evolution of the RCB specimen under the uniaxial loading process (**a**)RC-0°; (**b**)RCB-15°; (**c**)RCB-30°; (**d**)RCB-45°; (**e**)RCB-60°.

Infrared radiation is a direct consequence of the evolution and development of coal and rock defects and can reflect the damage degree of coal and rock. The development process of coal and rock cracks is the accumulation process of coal and rock damage. Figure 10 shows the infrared thermography of the inclined RCB specimen under UCT. The time when the inclined RCB specimen is obviously cracked is the stress is loaded to the post-peak stage (the post-peak stress σ/σ_p_ = 70%~80%). Cracks are mostly concentrated in the coal, coal and rock interface and near the bolt end, mainly the tensile cracks (Fig. 10(a–e)). Some shear cracks and tensile-shear cracks can also be observed during the loading process (Fig. 10(a–e)). Energy accumulation exists in coal and rock during loading, especially in coal (Fig. 10). During the test, the breaking sound can be heard clearly, and the fragments ejected after the burst damage can be observed. Therefore, each specimen has certain burst characteristics. Among them, the thermal image evolution of the 15°, 30° and 45° RCB specimens are especially obvious (Fig. 10(b–d)), and after the peak value, a strong burst failure occurred, causing the coal and rock to crack simultaneously. It is worth noting that the bolt can limit the possibility of sliding failure along the contact surface between the coal and rock. Before the impact failure occurs, the bolt played a role in inhibiting the accumulation of transverse deformation of the coal, making the accumulation of damage between the coal and the rock more unified. Finally, the failure form reflected that the coal and rock had a certain degree of cracking failure at the same time.

**Figure 11:**
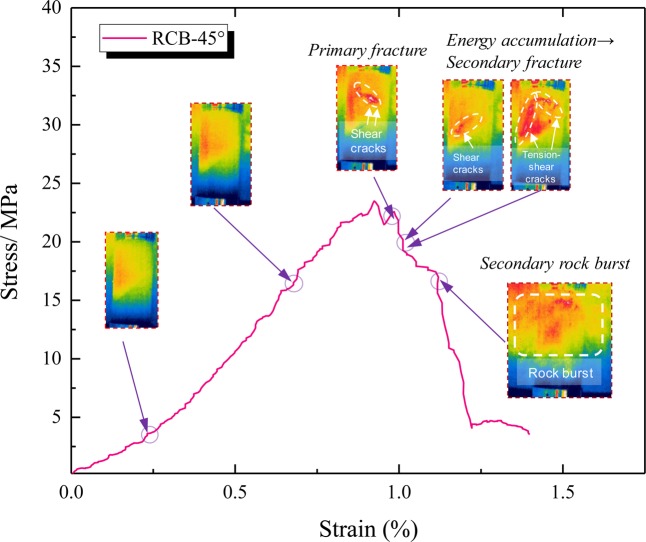
Typical secondary peak stress-strain curve and thermal imaging diagram of RCB-45 specimen.

Taking the RCB-45 specimen as an example, the thermal image evolution shows that the coal and rock experienced two burst failures during the loading process. The stress-strain curve and the corresponding thermal image process are illustrated in Fig. 11. When post-peak stress reaches σ/σ_p_ = 80%, the first shear crack is initiated and the initial burst failure is formed. With the continuous increase of stress, when the post-peak stress reaches σ/σ_p_ = 70%, the shear crack changes to tensile crack. The stress-strain curve rose slightly again and then formed a second drop. It should be pointed out that according to the evolution characteristics of the thermal infrared image, the stress-strain curve of the test shows two peaks. However, this does not imply that the specimen will finally be failure, and there may be a secondary burst. In other words, the cracks between the first and second damages are still further developed and accumulated, resulting in the final damages that may be stronger than the burst of the first peak fall. This kind of secondary burst failure has similar properties in the stress-strain curves of the RCB specimen in the UCT and TCT (Fig. 8, UCT: RC-0, RCB-15, RCB-45, RCB-60; TCT: RC-0, RCB-45, RCB-60).

### Temperature aging characteristics

**Figure 12:**
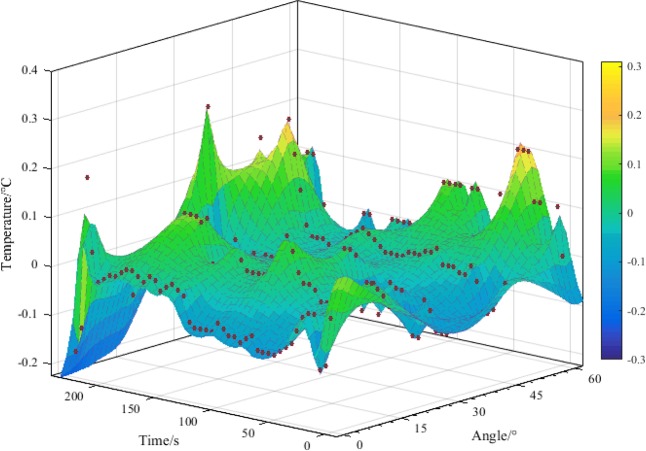
Temperature mutation characteristics of the RCB specimen (UCT).

During the failure process of the RCB specimen, primary and new cracks will release different degrees of infrared radiation. Specifically, shear cracks are distorted to generate heat, which will increase the temperature of infrared radiation. Tension cracks increase the volume of RCB specimen and absorb heat, which makes the temperature of infrared radiation drop. The average infrared radiation temperature (AIRT) was used to analyze the temperature field change in the failure area of the RCB specimen, and the results are shown in Fig. 12. Most of the specimen temperature abrupt points are in the post-peak stage, and there is a small fluctuation before the temperature abrupt point, which is slightly lower than 0 °C. It can be seen that after the RCB specimen is loaded, tension cracks are the main factors during the compaction and the accumulation of linear elasticity changes. When the cumulative damage reached the maximum value during impact failure, the temperature of the RCB specimen was both greater than 0 °C and less than 0 °C. Therefore, it can be seen that the RCB specimens have both shear and tensile failure.

## Analysis of burst characteristics of RCB specimen

### Analysis of stage stress parameters

Table 2 shows the stress parameter values of the specimens at each stage under UCT and TCT. The mechanical parameters of the RCB specimen have a definite correlation with the stress state and angle. The crack initiation stress σ_ci_ and the crack damage stress σ_cd_ of the rock are related to the rock properties and the internal crack distribution law and morphology, especially when the confining pressure is less than 30 MPa. Therefore, the influence of angle and confining pressure of the RCB specimen on burst tendency can be analyzed by comparing the crack initiation stress σ_ci_ and crack damage stress σ_cd_. Turichshev and Hadjigeorgiou^50^ proposed crack initiation stress index *C* and damage stress index *D* for layered rocks, as follows:

5 $$C=({\sigma }_{{\rm{ci}}}-{{\rm{P}}}_{{\rm{c}}})/({\sigma }_{{\rm{p}}}-{{\rm{P}}}_{{\rm{c}}})$$

6 $$D=({\sigma }_{{\rm{cd}}}-{{\rm{P}}}_{{\rm{c}}})/({\sigma }_{{\rm{p}}}-{{\rm{P}}}_{{\rm{c}}})$$

where, σ_ci_ is the crack initiation stress; σ_cd_ is the damage stress; σ_p_ is the peak stress; P_c_ confining pressure (P_c_ = 0 under the UCT).

**Table 2:** Mechanical parameters of specimens under UCT and TCT conditions (average value).

Conditions	Parameter	RC-0	RCB-15	RCB-30	RCB-45	RCB-60
UCT	Young’s modulus, *E* (GPa)	2.96	3.09	3.47	3.54	3.18
	UCS (MPa)	25.20	24.35	26.75	23.01	21.10
	Crack closure stress, σ_cc_ (MPa)	5.89	6.23	6.49	5.95	6.81
	Crack initiation stress, σ_ci_ (MPa)	10.20	11.61	13.03	9.72	10.93
	*C* = (σ_ci_ − P_c_)/(σ_p_ − P_c_)	0.40	0.48	0.49	0.42	0.52
	Crack damage stress, σ_cd_(MPa)	20.61	18.91	24.15	19.90	18.82
	*D* = (σ_cd_ − P_c_)/(σ_p_ − P_c_)	0.82	0.78	0.90	0.86	0.89
TCT	Young’s modulus, *E* (G*P*a)	15.29	18.88	12.46	24.69	25.12
	TCS (MPa)	73.80	37.94	47.81	58.53	27.85
	Crack closure stress, σ_cc_ (MPa)	13.80	12.93	22.81	16.10	9.36
	Crack initiation stress, σ_ci_ (MPa)	21.15	16.55	27.45	22.72	14.25
	*C* = (σ_ci_ − P_c_)/(σ_p_ − P_c_)	0.17	0.24	0.46	0.26	0.25
	Crack damage stress, σ_cd_(MPa)	63.25	31.61	42.10	51.00	24.25
	*D* = (σ_cd_ − P_c_)/(σ_p_ − P_c_)	0.83	0.78	0.85	0.85	0.82

**Figure 13:**
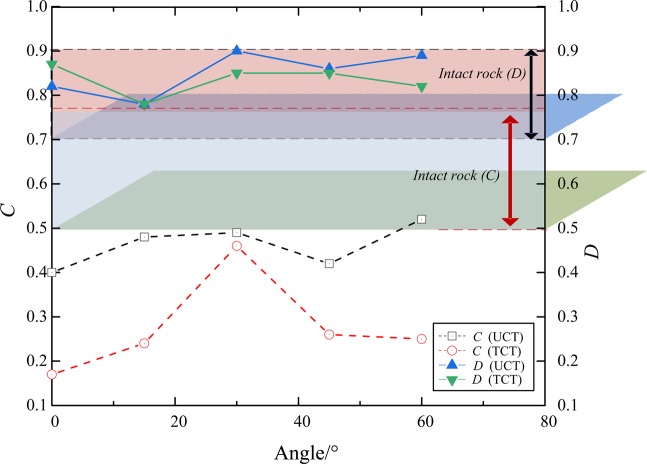
The relationship between the values of *C* and *D* for the inclined RCB specimen (under UCT and TCT conditions, compared with reference^55–62^).

Figure 13 shows the relationship between the values of *C* and *D* for the inclined RCB specimen (under UCT and TCT conditions, compared with references^55–62^). The *C* value of the specimen is within 0.40~0.52 (UCT), and the *C* value of the specimen is within 0.17-0.46 (TCT). The values of these two groups of test parameters are lower than the *C* values (0.5~0.78) of intact rock or specimen with inclined bedding^55–57^. Compared with the intact brittle rock, the RCB specimen will enter the stage of crack accumulation and development more easily and earlier under stress loading.

The *D* value of most intact brittle rocks is within 0.7~0.9^58–62^. In contrast, the value of specimen *D* is within 0.78~0.90 (UCT). Similar to intact brittle rock, the unstable crack propagation stage of the RCB specimen is completed in a short time under the UCT. The difference between crack damage stress σ_cd_ and UCS is small, and the RCB specimen under uniaxial loading will enter the post-peak stage faster. The *D* value of the triaxial loading specimen is lower than that of the uniaxial loading specimen except that of the RCB specimen. Generally, the confining pressure and the lateral restraining effect of the bolt will increase the unstable expansion stage of the RCB specimen, inhibit the development of cracks, and correspondingly stabilize the peak stress-strain to the unstable failure stage^63^. However, judging from the burst failure of the actual roadway engineering, as long as the cumulative value of failure stress under triaxial stress state is large enough, the possibility of induced failure of the external surrounding rock (i.e. the RCB system under uniaxial stress state) will be greater. This will make it easier to increase the possibility of burst tendency of the surrounding rock outside the roadway and may increase the coal burst zone.

### Comparison of burst index and stiffness coefficient

**Figure 14:**
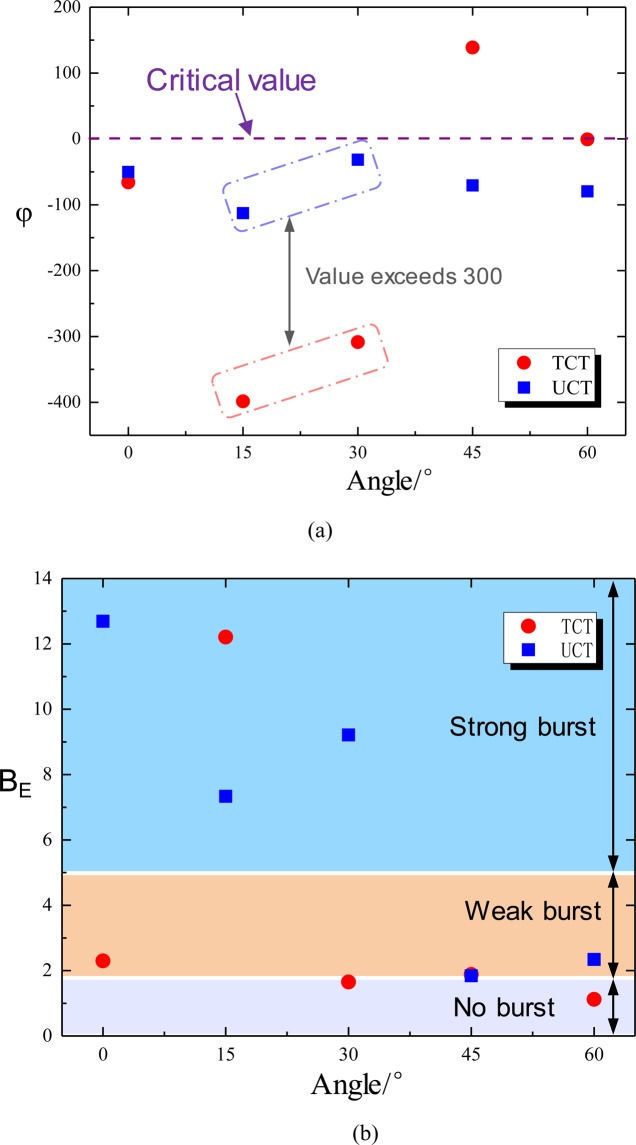
The relationship between the stiffness coefficient φ, the burst energy index B_E_ and the angle of the RCB specimen under UCT and TCT conditions (**a**) the stiffness coefficient φ and (**b**) the burst energy index B_E_.

As described in Section 2.2, when the sum of the stiffness coefficients of the RCB specimen is <0, the smaller the value is, the more obvious the burst tendency. The post-peak strain curve of the RCB specimen may drop two or more times (Fig. 8), as shown in the red dotted line on the right side of Fig. 3. If the drop stiffness coefficients *K*_rc_ are less than *K*_r_, this secondary burst failure tendency will be more serious. For burst-damaged rocks with multiple peaks, we can choose the smallest *K*_rc_ value to analyze. Figure 14(a) shows the law of stiffness coefficient φ and angle of the RCB specimen (under UCT and TCT conditions). The values φ of the specimens under UCT were all less than 0, indicating that the RCB specimen had different degrees of burst failure tendency under uniaxial loading. Among them, the RCB-15 specimen has the smallest value of −115, which is much smaller than other specimens (−30~−75), showing the greatest burst tendency. This is in accordance with the thermal image and the failure results of the samples observed in Section 4.3. Under the TCT, the φ values of 0°, 15°, and 30° angles are all less than 0, especially the RCB-30 specimen has a strong burst tendency. And the φ value reaches − 400, which is far lower than the value of RCB-30 specimen under UCT (−30).

The burst energy index *B*_E_ also shows that the RCB specimen has different degrees of burst damage under UCT and TCT (Fig. 14b). Under the UCT, the 0°, 15°, and 30° specimens have a strong burst tendency, and 45° and 60° specimens have a weak burst tendency. Under the TCT, the RCB-15 specimen indicates a strong burst tendency. In contrast, RCB-0°, 30°, and 45° specimens indicate weak burst tendency, and 60° specimen indicates no burst. It should be pointed out that the reason for the low tendency of burst at a large angle (60°) under triaxial stress may be that the coal and rock slip and fail along the combined surface without obvious burst tendency. When the angle is lower than 60°, the φ value of the TCT is much larger than the UCT, and the burst indexes decrease as the angle of the RCB specimen increases. This further illustrates that if a specimen tends to burst under the TCT, its burst tendency will be significantly greater than a specimen under UCT. Therefore, to some extent, the sum of stiffness coefficient φ and burst energy index *B*_E_ can reflect that the essence of burst failure of surrounding rock is the impact disturbance caused by the first instability of the internal rock. The burst tendency of the RCB-15 system is the largest under uniaxial and triaxial stress state. Furthermore, the zone of burst damage may also be affected by the instability of internal coal and rock mass.

## Conclusions

Based on the research background of the burst failure of the roadway engineering in thin coal seam mining under complex geological conditions, the burst tendency and mechanical behavior of the RCB specimen under different stress conditions are studied. The UCT and TCT of the RCB specimen with different angles are carried out, and the thermal image evolution analysis of the failure process of the UCT is also carried out. Furthermore, according to the established stiffness coefficient φ and burst energy index *B*_E_, the burst tendency of the RCB specimen in UCT and TCT is analyzed, and the following conclusions are obtained:

1. Compared with the intact brittle rock, the inclined RCB specimen is easier and earlier to enter into the crack accumulation and development. When the angle increases, the crack initiation stress σ_ci_ increases slightly. Similar to the intact brittle rock, the unstable crack growth stage of the RCB specimen is completed in a short time under UCT. The difference between the crack damage stress σ_cd_ and the UCS is small, and the RCB specimen will enter the post-peak stage faster. Under the triaxial stress condition, confining pressure and lateral restraint of bolt stabilize and increase the continuity of stress-strain progressive failure transition of the RCB specimen correspondingly. Thus the RCB specimen will enter the post-peak stage more slowly.
2. The stress-strain curve and thermal image evolution show that the peak of the stress-strain curve appears twice after the peak of some RCB specimens. The infrared thermal image evolutionary process is shown that the specimen is not fractured after the first peak. There may be a second burst. In other words, there are further cracks and energy accumulation between the first and second peak drops, and the final damage may be stronger than the burst of the first peak drop.
3. A burst tendency model and stiffness theory for RCB specimens is established. The sum of the stiffness coefficient *φ* can better describe the burst tendency of the RCB specimen. When the angle is less than 60°, the φ value and the burst energy index *B*_E_ of the TCT are significantly larger than those of the UCT. The burst tendency of the triaxial stress state RCB specimen is considerably greater than that under the uniaxial stress state. This explains that the essence of the rockburst damage on the surface of the roadway is due to the instability of the surrounding rock inside the roadway. Under the TCT and UCT conditions, the burst tendency is greatest when the combined angle is 15°. Additionally, the range of burst damage may also be affected by the instability of inside coal and rock masses.

## References

[1] Zuo JP (2017). Rock strata movement and subsidence based on MDDA, an improved discontinuous deformation analysis method in mining engineering. Arabian Journal of Geosciences.

[2] Weng L, Huang LQ, Taheri A, Li XB (2017). Rockburst characteristics and numerical simulation based on a strain energy density index: a case study of a roadway in linglong gold mine, China. Tunnelling and Underground Space Technology.

[3] Zhang JF, Jiang FX, Yang JB, Bai WS, Zhang L (2017). Rockburst mechanism in soft coal seam within deep coal mines. International Journal of Mining Science and Technology.

[4] Wang GF (2019). Rockburst mechanism and control in coal seam with both syncline and hard strata. Safety Science.

[5] Sun YJ, Zuo JP, Karakus M, Wang JT (2019). Investigation of movement and damage of integral overburden during shallow coal seam mining. International Journal of Rock Mechanics and Mining Sciences.

[6] Sun, Y. J., Zuo, J. P., Karakus, M. & Wen, J. H. A novel method for predicting movement and damage of overburden caused by shallow coal mining. *Rock Mechanics and Rock Engineering*. **53**, 1545–1563 (2020).

[7] Dou LM, Chen TJ, Gong SY, Hu H, Zhang ST (2012). Rockburst hazard determination by using computed tomography technology in deep workface. Safety Science.

[8] He MC, Nie W, Zhao ZY, Guo W (2012). Experimental investigation of bedding plane orientation on the rockburst behavior of sandstone. Rock Mechanics and Rock Engineering.

[9] Wang SY, Sloan SW, Sheng DC, Yang SQ, Tang CA (2014). Numerical study of failure behavior of pre-cracked rock specimens under conventional triaxial compression. International Journal of Solids and Structures.

[10] Lu CP (2015). Microseismic multi-parameter characteristics of rockburst hazard induced by hard roof fall and high stress concentration. International Journal of Rock Mechanics and Mining Sciences.

[11] He SQ (2019). Precursor of spatio-temporal evolution law of MS and AE activities for rock burst warning in steeply inclined and extremely thick coal seams under caving mining conditions. Rock Mechanics and Rock Engineering.

[12] Wu YZ, Gao FQ, Chen JY, He J (2019). Experimental study on the performance of rock bolts in coal burst-prone mines. Rock Mechanics and Rock Engineering.

[13] Cook NGW, Hoek EP, Pretorius JPG, Ortlepp WD, Salamon MDG (1966). Rock mechanics applied to the study of rockbursts. Journal- South African Institute of Mining and Metallurgy.

[14] Heunis R (1980). Development of rock-burst control strategies for south african gold mines. Journal- South African Institute of Mining and Metallurgy.

[15] Malan DF, Napier JAL (2018). Rockburst support in shallow-dipping tabular stopes at great depth. International Journal of Rock Mechanics and Mining Sciences.

[16] Carlsson A, Olsson T (1983). Rock bursting phenomena in a superficial rock mass in Southern Central Sweden. International Journal of Rock Mechanics & Mining Sciences & Geomechanics Abstracts.

[17] Hirata A, Kameoka Y, Hirano T (2007). Safety management based on detection of possible rock bursts by ae monitoring during tunnel excavation. Rock Mechanics and Rock Engineering.

[18] Wagner H (2019). Deep mining: a rock engineering challenge. Rock Mechanics and Rock Engineering.

[19] Chen BR, Feng XT, Li QP, Luo RZ, Li S (2013). Rock burst intensity classification based on the radiated energy with damage intensity at Jinping II hydropower station, China. Rock Mechanics and Rock Engineering.

[20] Cao AY (2016). Microseismic precursory characteristics of rock burst hazard in mining areas near a large residual coal pillar: a case study from Xuzhuang coal mine, Xuzhou, China. Rock Mechanics and Rock Engineering.

[21] Zuo JP (2017). Effects of thermal treatment on fracture characteristics of granite from Beishan, a possible high-level radioactive waste disposal site in China. Engineering Fracture Mechanics.

[22] Wu QH (2019). Experimental investigation of the dynamic response of prestressed rockbolt by using an SHPB-based rockbolt test system. Tunnelling and Underground Space Technology.

[23] Qin SQ, Jiao JJ, Tang CA, Li ZQ (2006). Instability leading to coal bumps and nonlinear evolutionary mechanisms for a coal-pillar-and-roof system. International Journal of Solids and Structures.

[24] Konicek P, Schreiber J (2018). Heavy rockbursts due to longwall mining near protective pillars:a case study. International Journal of Mining Science and Technology.

[25] Li PX, Feng XT, Feng GL, Xiao YY, Chen BR (2019). Rockburst and microseismic characteristics around lithological interfaces under different excavation directions in deep tunnels. Engineering Geology..

[26] He MC, Ren FQ, Liu D (2018). Rockburst mechanism research and its control. International Journal of Mining Science and Technology.

[27] Chen SJ, Yin DW, Jiang N, Wang F, Zhao ZH (2019). Mechanical properties of oil shale-coal composite samples. International Journal of Rock Mechanics and Mining Sciences.

[28] Zuo JP, Xie HP, Meng BB, Liu JF (2011). Experimental research on loading-unloading behavior of coal-rock combination bodies at different stress levels. Rock and Soil Mechanics.

[29] Zuo JP, Wang ZF, Zhou HW, Pei JL, Liu JF (2013). Failure behavior of a rock-coal-rock combined body with a weak coal interlayer. International Journal of Mining Science and Technology.

[30] Chen YL, Zuo JP, Liu DJ, Wang ZB (2018). Deformation failure characteristics of coal–rock combined body under uniaxial compression: experimental and numerical investigations. Bulletin of Engineering Geology and the Environment.

[31] Zhao ZH, Wang WM, Wang LH, Dai CQ (2015). Compression–shear strength criterion of coal–rock combination model considering interface effect. Tunnelling and Underground Space Technology.

[32] Huang BX, Liu JW (2013). The effect of loading rate on the behavior of samples composed of coal and rock. International Journal of Rock Mechanics and Mining Sciences.

[33] Gong FQ, Luo Y, Li XB, Si XF, Tao M (2018). Experimental simulation investigation on rockburst induced by spalling failure in deep circular tunnels. Tunnelling and Underground Space Technology.

[34] Gong FQ, Si XF, Li XB, Wang SY (2018). Experimental investigation of strain rockburst in circular caverns under deep three-dimensional high-stress conditions. Rock Mechanics and Rock Engineering.

[35] Nguyen N, Oehlers D, Bradford M (2001). An analytical model for reinforced concrete beams with bolted side plates accounting for longitudinal and transverse partial interaction. International Journal of Solids and Structures.

[36] Whitney TJ, Iarve EV, Brockman RA (2004). Singular stress fields near contact boundaries in a composite bolted joint. International Journal of Solids and Structures.

[37] Li CC (2010). Field observations of rock bolts in high stress rock masses. Rock Mechanics & Rock Engineering.

[38] Cai M (2013). Principles of rock support in burst-prone ground. Tunnelling and Underground Space Technology.

[39] Wu QH (2019). Experimental investigation on rockbolt performance under the tension load. Rock Mechanics and Rock Engineering.

[40] Zuo JP (2019). Investigation on the interaction mechanism and failure behavior between bolt and rock-like mass. Tunnelling and Underground Space Technology.

[41] Vardoulakis I, Labuz JF, Papamichos E, Tronvoll J (1998). Continuum fracture mechanics of uniaxial compression on brittle rocks. International Journal of Solids and Structures.

[42] Zhou XP, Zhang YX, Ha QL, Zhu KS (2004). Bounds on the complete stress–strain relation for a crack-weakened rock mass under compressive loads. International Journal of Solids and Structures.

[43] Francesco P (2019). Experimental characterization and numerical modelling of fracture processes in granite. International Journal of Solids and Structures.

[44] Zuo, J.P., Wang J.T., & Jiang, Y. Q. Macro/meso failure behavior of surrounding rock in deep roadway and its control technology. *International Journal of Coal Science & Technology* 6.3:301–319 (2019).

[45] Li DQ (2014). Mining thin sub-layer as self-protective coal seam to reduce the danger of coal and gas outburst. Natural Hazards.

[46] Li M, Lu J, Xiong S (2019). Prediction of Fractures in Coal Seams with Multi-component Seismic Data. Scientific Reports.

[47] Zhao D, Wu Q (2018). An approach to predict the height of fractured water-conducting zone of coal roof strata using random forest regression. Scientific Reports.

[48] Li QL, Peng SP, Zou GG (2015). High resolution processing of 3D seismic data for thin coal seam in Guqiao coal mine. Journal of Applied Geophysics.

[49] Wu C (2019). A new seismic-based strain energy methodology for coal burst forecasting in underground coal mines. International Journal of Rock Mechanics and Mining Sciences.

[50] Kidybiński. A (1981). Bursting liability indices of coal. International Journal of Rock Mechanics & Mining Science & Geomechanics Abstracts.

[51] Zhou J, Li XB, Mitri HS (2018). Evaluation method of rockburst: state-of-the-art literature review. Tunnelling and Underground Space Technology.

[52] Wu G.S., Yu W.J., Liu Z. & Tang Z. Failure law and mechanism of the rock-loose coal specimen under combined loading rate. *Advances in Civil Engineering*. 2018, Article ID 2482903, 10 pages

[53] Zuo, J. P. *et al*. Experimental study of the ultrasonic and mechanical properties of a naturally fractured limestone. *International Journal of Rock Mechanics and Mining Sciences* **125**, 104162, 10.1016/j.ijrmms.2019.104162 (2020).

[54] Fairhurst CE, Hudson JA (1999). Draft isrm suggested method for the complete stress-strain curve for intact rock in uniaxial compression. International Journal of Rock Mechanics & Mining Science & Geomechanics Abstracts.

[55] Turichshev A, Hadjigeorgiou J (2016). Triaxial compression experiments on intact veined andesite. International Journal of Rock Mechanics and Mining Sciences.

[56] Fonseka GM, Murrell SAF, Barnes P (1985). Scanning electron microscope and acoustic emission studies of crack development in rocks. International Journal of Rock Mechanics and Mining Sciences &, Geomechanics Abstracts.

[57] Bieniawski ZT (1967). Mechanism of brittle fracture of rock, Parts I, II and III. International Journal of Rock Mechanics and Mining Sciences &, Geomechanics Abstracts.

[58] Martin CD. The strength of massive Lac du Bonnet granite around underground opening. PhD thesis, University of Manitoba, 1993.

[59] Hatzor YH, Palchik V (1997). The influence of grain size and porosity on crack initiation stress and critical flaw length in dolomites. International Journal of Rock Mechanics and Mining Sciences.

[60] Eberhardt E, Stead D, Stimpson B (1999). Quantifying progressive pre-peak brittle fracture damage in rock during uniaxial compression. International Journal of Rock Mechanics and Mining Sciences.

[61] Fakhimi A, Carvalho F, Ishida T, Labuz JF (2002). Simulation of failure around a circular opening in rock. International Journal of Rock Mechanics & Mining Sciences.

[62] Cai M (2004). Generalized crack initiation and crack damage stress thresholds of brittle rock masses near underground excavations. International Journal of Rock Mechanics & Mining Sciences.

[63] Yu WJ, Pan B, Zhang F, Yao SF (2019). Deformation characteristics and determination of optimum supporting time of alteration rock mass in deep mine. KSCE Journal of Civil Engineering.

